# A Case Report of Neonatal Supraventricular Tachycardia Resolved with Single-Syringe Adenosine

**DOI:** 10.5811/cpcem.2020.9.48829

**Published:** 2020-10-20

**Authors:** Marc McDowell, Tasneem Ahmed, Bill Schroeder, Shannon Staley

**Affiliations:** Advocate Children’s Hospital, Department of Emergency Medicine, Oak Lawn, Illinois

**Keywords:** Supraventricular tachycardia, adenosine, SVT, neonatal

## Abstract

**Introduction:**

Supraventricular tachycardia (SVT) is a condition requiring emergency care in neonates.

**Case Report:**

We describe a successfully treated case of neonatal SVT in a four-week-old neonate using the novel adenosine administration method. This technique is potentially easier to facilitate and does not require equipment such as a stopcock. Adenosine 0.2 milligrams per kilogram was drawn up into a syringe containing 0.9% sodium chloride to a total volume of 3 milliliters. Once administered, the patient had near-immediate return to normal sinus rhythm without sequelae.

**Conclusion:**

This case demonstrates that the single-syringe method appears potentially safe and effective in neonates.

## INTRODUCTION

Supraventricular tachycardia (SVT) is a tachydysrhymia not uncommonly seen in the emergency department (ED). This acute disease state accounts for approximately 50,000 ED visits annually.^1^,^2^ A recent population-based study from a national, birth cohort database in Taiwan of children born between 2000 and 2008 with complete postnatal medical data estimated the cumulative incidence of SVT in patients aged less than one month as 0.06 per 1000 patient-years overall and 0.05 per 1000 patient-years in those without major congenital heart disease.^3^ Supraventricular tachycardia is a comprehensive term for a number of rhythms that range from benign sinus tachycardia to Wolff-Parkinson-White syndrome.^4^

Adenosine, a naturally occurring purine nucleoside, is formed from the breakdown of adenosine triphosphate. It acts through a cascade of secondary messengers that initially block atrioventricular nodal conduction via the A1 receptors in the cardiac tissue. These in turn act on Gi proteins, which decrease cyclic adenosine monophosphate. This leads to stimulation of potassium channels and inhibition of L-type calcium channels, causing hyperpolarization of cardiac myocytes, thus returning the heart to a normal sinus rhythm (NSR).^5^

Given adenosine’s unique pharmacokinetic profile of near-immediate onset and a half-life of 5–10 seconds, cardioversion can be performed rapidly with limited adverse effects. Due to its rapid metabolism, a flush of saline is also administered to ensure adequate delivery to the myocardium. The American Heart Association Pediatric Advanced Life Support guidelines recommend adenosine after a trial of vagal maneuvers.^6^ Adenosine is commonly administered as a 0.1 milligrams (mg) per kilogram (kg) (maximum 6 mg) rapid intravenous (IV) bolus over one to two seconds followed by a rapid 5–10 milliliter (mL) saline flush. If the first dose does not result in termination of SVT to NSR within one to two minutes, a repeat dose of 0.2 mg/kg (maximum 12 mg) can be given and the dose may be repeated one additional time if required (for a total of three doses).^7^

To facilitate administering drug and saline flush a three-way stopcock is commonly used; however, this method has physical logistics issues. For example, locating a three-way stopcock may cause a delay in therapy and certain emergency medical transport services may not stock such a device.^8^ An alternative method of combining the drug and the flush in a single-syringe may offer higher rates of conversion, lower need for repeat dosing, eliminate the need for a stopcock or additional staff, and limit peripheral IV extravasations. Combining medication and flush has been popularized by Free Open Access Medical Education (FOAM) in recent years. In 2019 McDowell et al conducted a prospective, observational cohort study demonstrating the dilute, single-syringe method as non-inferior to the conventional two-syringe method in adults.^9^

## CASE REPORT

**Image 1:**
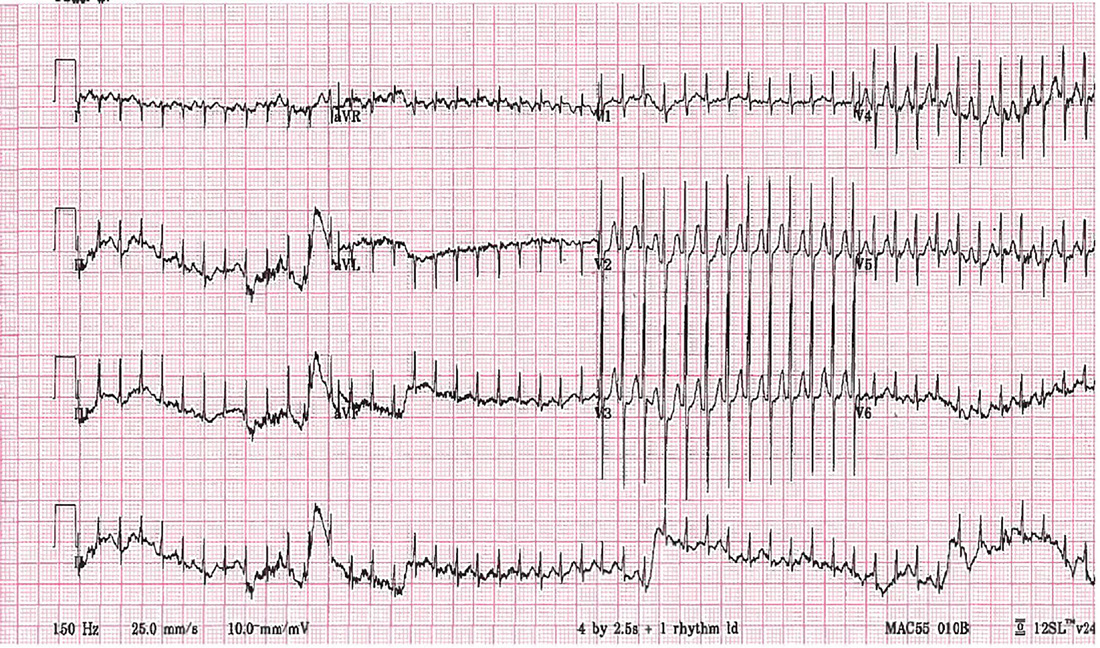
Narrow complex tachydysrhythmia in four-week-old patient.

A full-term, four-week-old male with past medical history of supraventricular tachycardia presented to the ED with a heart rate of 299 beats per minute. An electrocardiogram (ECG) demonstrated a rapid, narrow-complex tachyarrhythmia determined to be SVT (Image 1).

**Image 2:**
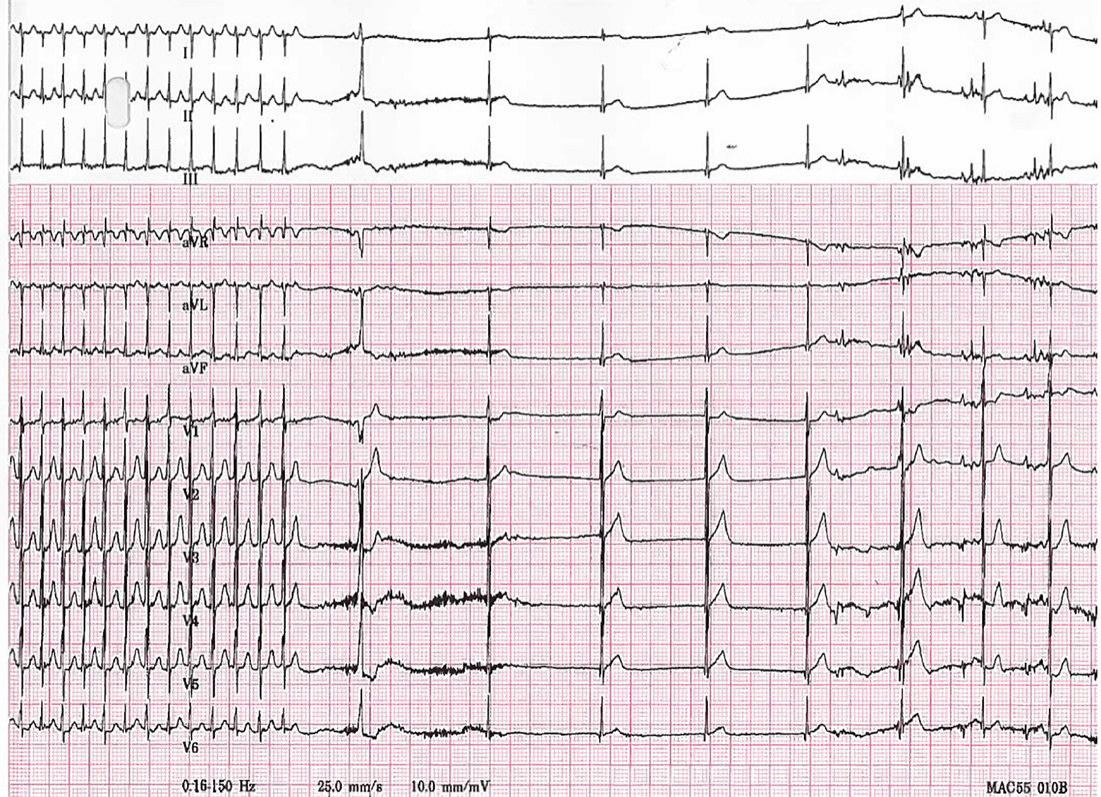
Electrocardiogram during administration of 0.2 milligrams per kilogram of adenosine via single-syringe method.

The patient was well perfused with a blood pressure of 62/54 millimeters of mercury (mm Hg). However, oxygen saturation was poor at 82% on 3 liters per minute via nasal cannula and a respiratory rate of 32 breaths per minute. Due to the immediacy of the illness, pharmacologic cardioversion was selected over non-invasive vagal maneuvers. Obtaining supplies to initiate a mammalian diving reflex or facilitating a modified valsalva maneuver were deemed to require too much time. A dose of 0.2 mg/kg of adenosine was prepared by the pharmacist and mixed in a single syringe with 0.9% sodium chloride to a total of 3 mL. The adenosine was administered as a rapid IV push via 24-gauge antecubital IV (Image 2).

**Image 3:**
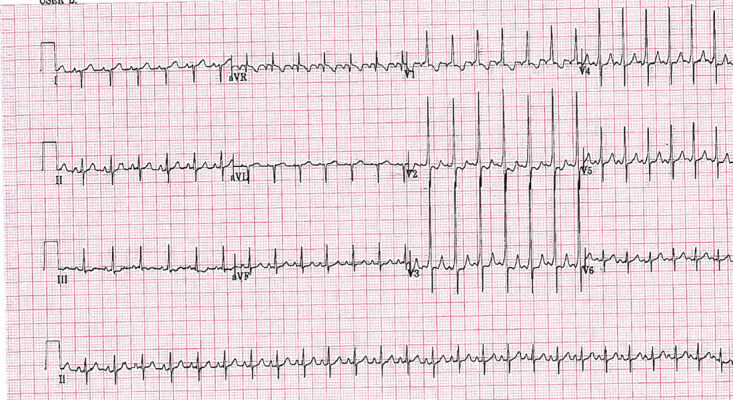
Return to sustained normal sinus rhythm with short PR interval and apparent delta waves indicating Wolff-Parkinson-White syndrome.

The arrhythmia terminated and the patient returned to sinus rhythm with a heart rate of 166 beats per minute (Image 3). Of note, shortened PR intervals with slurred upstrokes resembling delta waves were observed in leads V2–V5. This is reminiscent of Wolff-Parkinson-White syndrome. Pediatric cardiology noted this to be definite evidence of a pre-excitation rhythm.

CPC-EM Capsule

What do we already know about this clinical entity?

*Supraventricular tachycardia is a life-threatening tachydysrhymia. Adenosine is an effective treatment, but administration can be cumbersome*.

What makes this presentation of disease reportable?

*We report a novel method of adenosine administration, not previously reported in the neonatal population*.

What is the major learning point?

*Single-syringe diluted adenosine was safely and effectively administered in a 4-week-old neonatal patient in the emergency department*.

How might this improve emergency medicine practice?

*Providers may consider the transition from traditional administration to single-syringe, diluted method if clinically appropriate*.

Blood pressure and oxygen saturation both returned to normal limits, 86/73 mm Hg and 99%, respectively. Neither the nurse, pharmacist, nor physician had any difficulty with their tasks and noted the ease of the single-syringe method over the traditional two-syringe method. Point-of-care echocardiogram obtained immediately following conversion to sinus rhythm showed subjectively normal function. The patient was transferred to the pediatric cardiac intensive care unit and maintained normal sinus rhythm for the remainder of his admission.

## DISCUSSION

We describe a case of neonatal SVT presenting in respiratory distress with return to NSR with the diluted single-syringe method of adenosine. Adenosine is an effective, guideline-recommended treatment that can chemically convert SVT. The diluted single-syringe method of adenosine is effective in the treatment of adult SVT. Neonatal SVT was safely and effectively treated in this patient. If materials, time, or personnel are limited, the single-syringe method can be considered.

## CONCLUSION

Single-syringe adenosine is relatively easier to compound with available materials readily found in an ED. Utilization of diluted adenosine appears potentially safe and effective when administered in the neonatal population for the treatment of supraventricular tachycardia.
